# Epha3 acts as proangiogenic factor in multiple myeloma

**DOI:** 10.18632/oncotarget.16100

**Published:** 2017-03-10

**Authors:** Antonella Caivano, Francesco La Rocca, Ilaria Laurenzana, Tiziana Annese, Roberto Tamma, Ubaldo Famigliari, Vittorio Simeon, Stefania Trino, Luciana De Luca, Oreste Villani, Simona Berardi, Antonio Basile, Angelo Vacca, Giuseppe Saglio, Luigi Del Vecchio, Pellegrino Musto, Daniela Cilloni

**Affiliations:** ^1^ Laboratory of Pre-clinical and Translational Research, Scientific Institute of Research and Cure (IRCCS), Referral Cancer Center of Basilicata (CROB), Rionero in Vulture, Italy; ^2^ Department of Human Anatomy, Histology and Embryology, University of Bari Medical School, Bari, Italy; ^3^ Division of Pathology, Department of Oncology, St Luigi Hospital, Turin, Italy; ^4^ Department of Onco-Hematology, IRCCS-CROB, Rionero in Vulture, Italy; ^5^ Department of Biomedical Sciences and Human Oncology, University of Bari Medical School, Bari, Italy; ^6^ Department of Clinical and Biological Sciences, University of Turin, Orbassano, Italy; ^7^ CEINGE-Biotecnologie Avanzate s.c.a r.l and Medical Biotechnologies, Federico II University, Naples, Italy; ^8^ Department of Molecular Medicine and Medical Biotechnologies, Federico II University, Naples, Italy; ^9^ Scientific Direction, IRCCS-CROB, Rionero in Vulture, Italy

**Keywords:** angiogenesis, bone marrow endothelial cells, receptor tyrosine kinase, EphA3, multiple myeloma

## Abstract

This study investigates the role of ephrin receptor A3 (EphA3) in the angiogenesis of Multiple Myeloma (MM) and the effects of a selective target of EphA3 by a specific monoclonal antibody on primary bone marrow endothelial cells (ECs) of MM patients.

EphA3 mRNA and protein were evaluated in ECs of MM patients (MMECs), in ECs of patients with monoclonal gammopathies of undetermined significance (MGECs) and in ECs of healthy subjects (control ECs). The effects of EphA3 targeting by mRNA silencing (siRNA) or by the anti EphA3 antibody on the angiogenesis were evaluated. We found that EphA3 is highly expressed in MMECs compared to the other EC types. Loss of function of EphA3 by siRNA significantly inhibited the ability of MMECs to adhere to fibronectin, to migrate and to form tube like structures *in vitro*, without affecting cell proliferation or viability. In addition, gene expression profiling showed that knockdown of EphA3 down modulated some molecules that regulate adhesion, migration and invasion processes. Interestingly, EphA3 targeting by an anti EphA3 antibody reduced all the MMEC angiogenesis-related functions *in vitro*. In conclusion, our findings suggest that EphA3 plays an important role in MM angiogenesis.

## INTRODUCTION

Multiple myeloma (MM) is a plasma cell (PC) clonal disorder which originates from post germinal center B cells that accumulate somatic hypermutation and immunoglobulin heavy-chain class switching. PCs typically locate in the bone marrow which is crucial for MM cell growth and survival [1]. Particularly, angiogenesis critically partecipates to pathophysiology and progression of MM [2]. Despite availability of several new therapeutic agents, MM is incurable for most patients [3]. Therefore, it need to develop and find new agents targeting additional pathways relevant for MM cells maintenance to increase the spectrum of available therapies. In this scenario, monoclonal antibodies against MM cell antigens represent a possible therapeutic approach [3]. However, their application is compromised by a lack of appropriate antigen targets on MM cell surface.

The ephrins (Efn) and their receptors (Eph) have recently emerged as possible therapeutic targets [4], since they control pathways which are critical for the development and maturation of myeloid and lymphoid cells [5, 6]. The Ephs, a large family of receptor tyrosine kinases, were divided in two subfamilies: the EphA and EphB, according to the preferential ligand. Usually, EphA receptors interact with glycophosphatidylinositol (GPI)-anchored EfnAs, and EphB interact with transmembrane EfnBs, although there is exception to this [7]. Eph receptors are involved in different biological process such as cell adhesion, migration and axon guidance, during development and homeostasis of many tissues [8–12]. Moreover, recent studies demonstrate that Eph-Efn signaling has important roles in cancer growth, progression and angiogenesis [13].

The overexpression of EphA3 has been demonstrated in different cancers, such as lung cancers, melanomas, gastric carcinoma, leukemia [14–17], in B and T cell malignancies [18, 19] and in glioblastoma multiforme [20]. EphA3 was important in angiogenesis and prognosis of gastric and pancreatic carcinoma [17]. Recently, Vail et al. demonstrated that EphA3 was overexpressed in microenvironment of some human cancers and mouse tumor xenografts. EphA3 was found on mouse bone marrow mesenchymal and myeloid cells. Mice treated with an anti-EphA3 antibody showed a reduction of tumor growth and a destruction of tumor stroma and vasculature [21].

Based on the original anti EphA3 monoclonal antibody IIIA4 [14, 22], a modified IgG1 antibody against EphA3 (KB004) was generated and it is under phase 1/2 clinical trials for the treatment of EphA3 over-expressing hematological myeloid malignancies refractory to conventional treatment [23].

No data are available in literature regarding the EphA3 expression in MM patients and its role in developing or sustaining the MM malignant cell growth, in inducing progression and in the angiogenesis in BM microenvironment.

Here we reported for the first time the proof of concept of the antiangiogenic activity of specific antibody anti human EphA3 showing a potent anti-angiogenic activity *in vitro* which might have potential therapeutic applications.

## RESULTS

### EphA3 is upregulated in MMECs vs MGECs and normal ECs

**Figure 1:**
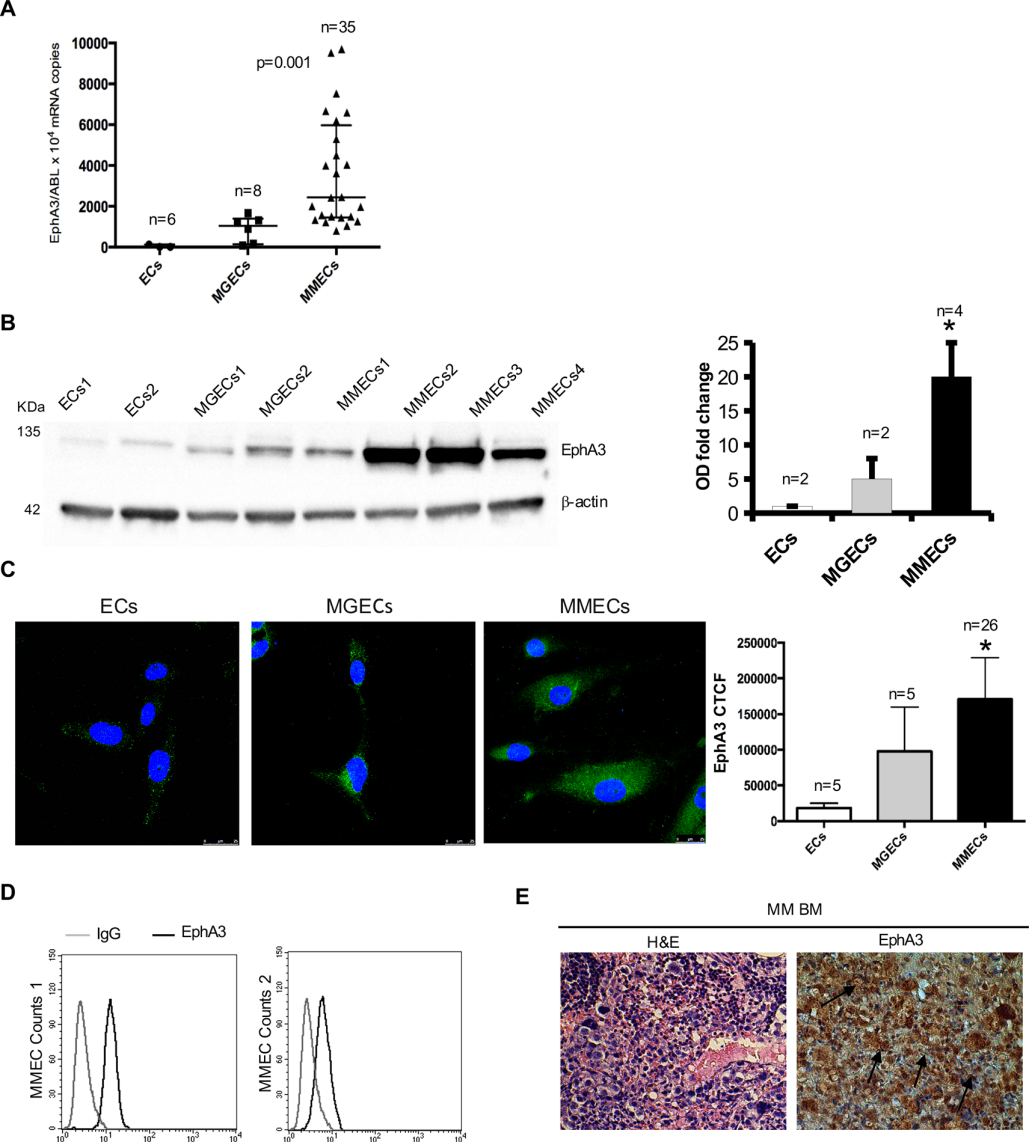
Analysis of EphA3 expression in normal ECs vs MGECs vs MMECs (**A**) Absolute Real Time-PCR of EphA3 mRNA copies/10*4 *ABL* copies as median ± SD of 6 normal (●) and 8 MGUS (■) and 35 MM subjects (▲) respectively. *p* = 0.001 by One Way ANOVA and Kruskal-Wallis test. (**B**) Western blot of representative 2 normal, 2 MGUS and 4 MM subjects (β-acti*n* = loading control). EphA3 fold change of Optical Density (OD) as means ± SD of 2 normal, 2 MGUS and 4 MM subjects. (**C**) Confocal immunofluorescence of EphA3 in MMECs *vs*. MGECs *vs*. ECs. Corrected Total Cell Fluorescence (CTCF) as mean ± SD of 26 MM and 5 MGUS and 5 normal subjects. Pictures by confocal laser scanning microscope with 40× objective lenses. *p < 0.03 or better by Wilcoxon signed-rank. (**D**) FACS analysis of EphA3 protein expression in MMECs from 2 representative patients. (**E**) EphA3 immunohistochemical staining of BM biopsies from representative MM patient. EphA3 stained both neovessels (arrows) and plasma cells (arrows). Hematossilin/eosin staining (H&E) of BM biopsies is showed as magnification of 40×. Pictures by an Olympus photomicroscope (Olympus, Milan, Italy) with a CCD camera (Princeton Scientific Instr., Princeton, NJ, USA).

In order to sudy EphA3 expression in MM, we measured messenger RNA (mRNA) and protein levels in primary normal ECs, MGECs, and MMECs. Absolute quantitative real-time-PCR was performed on these ECs. EphA3 mRNA amount increased from ECs to MGECs, reaching the highest levels in MMECs (Figure 1A). Furthermore, we observed a trend of increased EphA3 expression in ECs based on MM stage (Supplementary Figure 1). Western blot and immunofluorescence showed over-expression of EphA3 protein among the different EC types (Figure 1B–1C). EphA3 protein expression in MMECs was confirmed using flow cytometry and representative expression profiles are shown in Figure 1D. An intense and diffuse EphA3staining were observed on MM micro vessels and PCs in MM BM biopsies (Figure 1E).

### Loss of EphA3 inhibited angiogenesis *in vitro*

**Figure 2:**
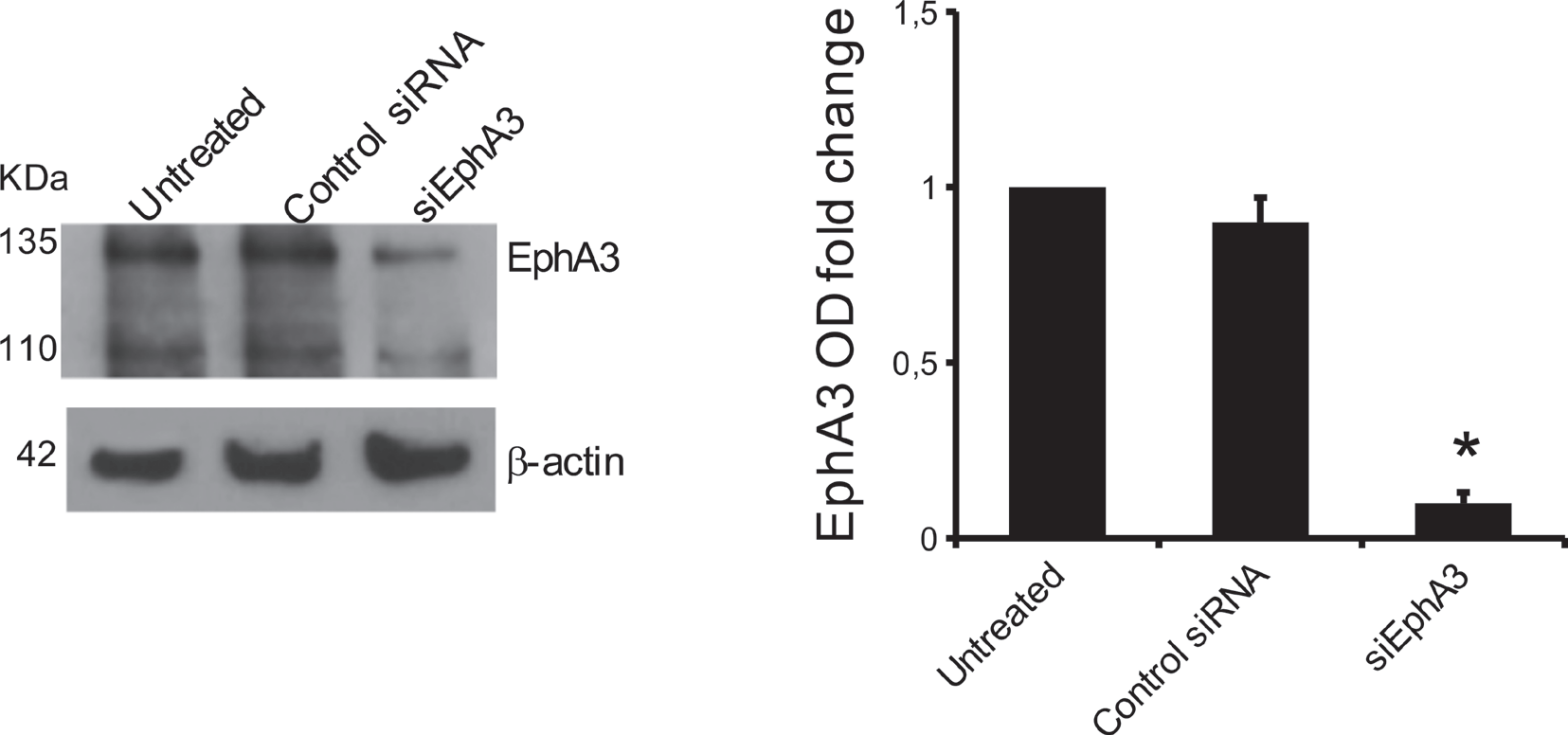
EphA3 silencing in MMECs The cells were transfected with EphA3 siRNA (siEphA3), non-targeting siRNA (Control siRNA) or lipofectamine only (Untreated) and analyzed after transfection in a Western blot assay (β-acti*n* = loading control). In the left panel, WB of a representative MMEC sample was showed. Data are means ± SD of 10 MM patients. *p < 0.03

**Figure 3:**
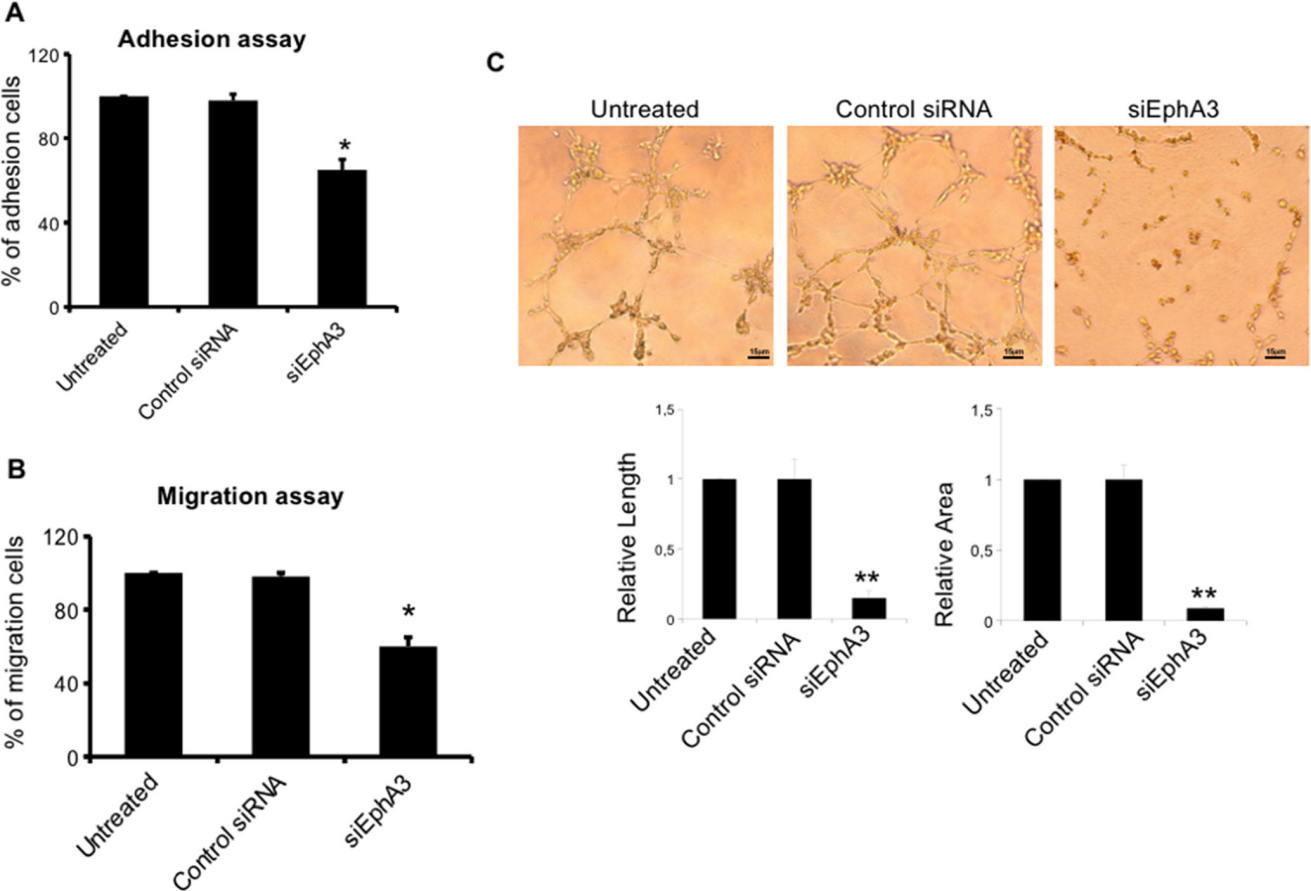
Effects on EC functions and angiogenesis in siEphA3 MMECs (siEphA3) siRNA-transfected cells were tested for adhesion to fibronectin (**A**), chemotaxis (**B**) and angiogenesis on Matrigel (**C**; quantification by vessel length and areas in the bottom panels) and compared with control siRNA and untreated cells by the EVOS image software. Matrigel original magnification ×200 for all panels. Data are means ± SD of 8 MM patients. *p < 0.03 or better and **p < 0.01 or better by Wilcoxon signed-rank test.

To define the role of EphA3 in MMEC angiogenesis, we knocked down its gene by mRNA silencing (siRNA). In siEphA3-MMECs the protein was reduced by over 80% *vs* untreated or non-targeted siRNA cells (Control siRNA MMECs; Figure 2). EphA3-siRNA did not affect cell viability nor induce apoptosis (data not shown), but affected cell adhesion (–35%) and chemotaxis (–40%) (Figure 3A–3B). The siEphA3-MMECs plated on the Matrigel (which mimics the sub endothelial basement membrane) gave no angiogenesis, i.e. it showed a significant reduction in the vessel areas and length (Figure 3C). Moreover, we demonstrated that EphA3 silencing did not affect angiogenic functions of MGECs and normal ECs *in vitro* (Supplementary Figure 2).

Moreover, we correlated the number of EphA3 copies (range from 850 to 9500 EphA3 copies) to the relative length of the arms and the relative area of Matrigel assay in ECs from 10 MM patients by Pearson correlation coefficient (PCC) analysis. We observed a PCC between length of the arms or area of Matrigel and number of copies of EphA3 of 0.647 (*p* = 0.04) and 0.598 (*p* = 0.06) respectively. It indicated a significant positive and good correlation between EphA3 and length and a positive and moderate correlation between EphA3 and area.

### EphA3 knockdown modulated molecules of adhesion, migration and invasion processes

**Figure 4:**
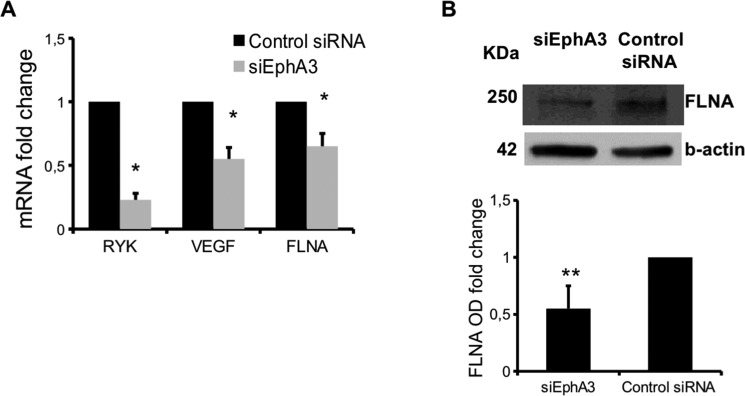
mRNA and proteins differentially expressed in siEphA3 vs Control siRNA cells Relative quantitative real time-PCR (normalized to Abelson = ABL) was performed for RYK, VEGF, and FLNA mRNA (**A**). FLNA protein expression was evaluated by western blot (**B**) β-actin = loading control). Optical density (OD) as means ± SD of 3 independent experiments. *p < 0.03 and **p < 0.01 by Wilcoxon signed-rank test.

The transcriptional profiles of EphA3-siRNA MMECs were compared with those of non-targeted siRNA cells by gene expression profiling analysis. Among the significantly modulated genes [(190 genes (Supplementary Table 1 and Supplementary Figure 3)], we found downregulation of the trafficking and angiogenesis molecules such as Receptor-Like Tyrosine Kinase *(RYK)*, Junctional Adhesion Molecule 2 *(JAM2)*, Vascular Endothelial Growth Factor A *(VEGFA)*, Filamin A *(FLNA)*, CD248 in EphA3 siRNA MMECs (Table 1). To validate the differential expression of genes, real time PCR analysis was performed in siEphA3 and Control siRNA MMECs. We confirmed the down-regulation of RYK, VEGF and FLNA mRNA in siEphA3 vs Control siRNA cells (Figure 4A). Moreover, low FLNA protein levels were reported in siEphA3 MMECs when compared to Control siRNA MMECs (Figure 4B).

**Table 1:** Down regulated genes in siEphA3 *vs* control siRNA MMECs

ENTREZ GENESYMBOL ID	NAME	FUNCTION	FOLD CHANGE
57124	**CD248/TEM1**	Angiogenesis^35^	0,8
2316	**FLNA**	Adhesion, migration^34^	0,8
7422	**VEGFA**	Angiogenesis^36^	0,7
58494	**JAM2**	Cell-cell adhesion^37^	0,7
6259	**RYK**	Focal adhesion^38^	0,3

### EphA3-specific antibody inhibited MMECs *in vitro* migration and tube formation

**Figure 5:**
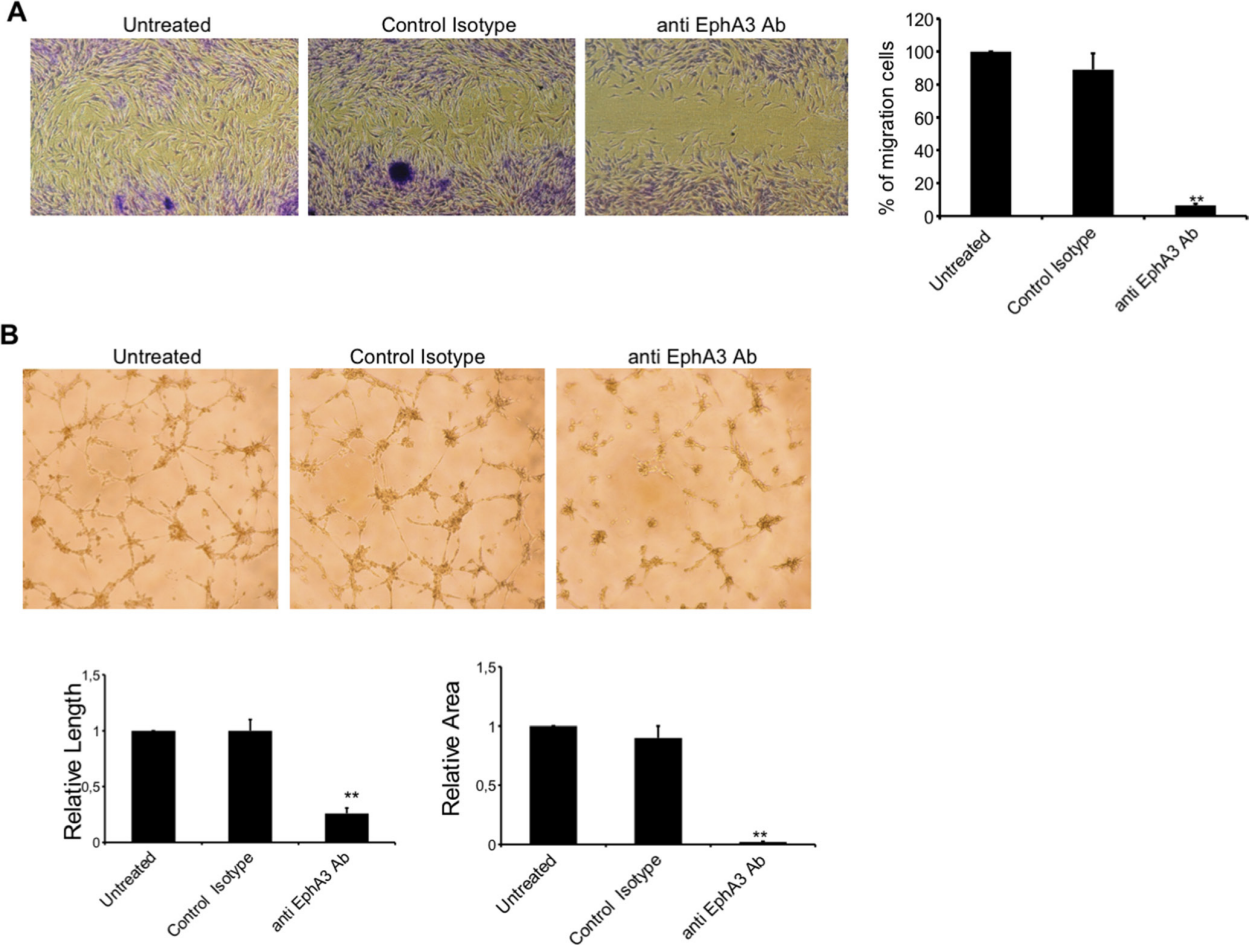
Characterization of anti angiogenic capability of anti EphA3-specific antibody (**A**) Analysis of migration of MMECs by wound healing assays *in vitro*. Representative photographs (4X magnification) taken 24h after scratching are shown. Cell migration was monitored over 24h with 7.5 μg/ml of anti EphA3-Ab or an irrelevant Ab (Control) or in medium (Untreated) as indicated. (**B**) MMECs were cultured on standard Matrigel in the absence (Untreated) or presence of anti-EphA3 or an irrelevant Ab (Control). Representative microphotographs of tube formation after 18 hours of culture (original magnification ×4) are shown. Quantification of lateral migration and tube formation (vessel length and areas) is shown at right panels. Anti EphA3 was assayed at least three times and the corresponding values (means ± SD) of 4 MM patients were represented, **p < 0.01 versus control.

To examine whether anti-EphA3 Ab (chIIIA4) affected the angiogenic functions of MMECs, we performed wound healing and tube formation assays. First, MMECs treated with anti EphA3 were not affected for viability and apoptosis (data not shown). Wound healing assay showed a rate of migrated MMECs from ≈ 90% (control) to 15% in the presence of anti-EphA3 Ab (chIIIA4). As expected, MMEC migration was not impacted by an isotypic Ab (Figure 5A). MMECs treatment with anti-EphA3 Ab (chIIIA4) significantly reduced the formation of tube-like structures (Figure 5B). Overall, these data showed significant, *in vitro*, evidence of the antiangiogenic activity of anti EphA3, affecting EC migration and tubulogenesis.

## DISCUSSION

BM microenvironment supports survival and progression of MM cells. The activation/generation of ECs and the consequent angiogenesis seems to be crucial in this process. The microenvironment consists of a ‘niche’ of BM stromal cells (BMSCs), such as fibroblasts, osteoclasts, osteoblasts, vascular endothelial cells, lymphocytes and extracellular matrix (ECM). The crosstalk between MM cells and the BM niche, mediated by cytokines and adhesion molecules, is critical for the trafficking of neoplastic cells to the BM, for production of tumor survival factors and for inhibition of osteoblastogenesis. Several novel agents including proteasome inhibitors (i.e. bortezomib) and immunomodulatory drugs (ImiDs; I.e, lenalidomide) have revolutionized the treatment of MM resulting in a significant improvement of the overall survival; their activity is also through an anti-angiogenetic effect [24, 25]. Complete remission can be achieved but recurrence of the disease remains the main obstacle to cure. Considering the critical role of angiogenesis in sustaining the survival and proliferation of MM cells, there is a need of more effective drugs targeting the microenvironment, and particularly the angiogenesis, to improve the clinical outcome for MM patients.

Eph receptors and their membrane-bound ephrin ligands are involved in many biological processes including adhesion, cell migration and angiogenesis. Vascular endothelium is positive for Efn-B2 and the Eph receptors EphB3 and EphB4. Moreover, Efn-B2 and EphB4 are expressed on arteries and veins, respectively, during embryonic development and are required for vascular remodeling during vessel maturation [26]. Furthermore, the Efn-A1 and its EphA2 receptor are expressed in tumor angiogenesis [27]. Interestingly, the angiogenesis was compromised by inhibiting Eph/ephrin signaling using specific anti Eph/Efn antibodies [21].

EphA3 receptor plays critical role in many solid tumors as well as hematological malignancies [28–30]. Recently, Vail and colleagues reported that microenvironment of different human cancers and mouse tumor xenografts were positive for EphA3 expression. Its expression in many human tumors and not in normal tissues, together with antitumor properties of the anti EphA3mAb (chIIIA4), defined EphA3 as potential target for antibody-based anticancer therapies [21].

Moreover, both tumor growth and angiogenesis were inhibited *in vivo* using soluble EphA2-Fc and EphA3-Fc constructs [31]. This demonstrated that EphA3 receptor had a key role in the regulation of tumor angiogenesis.

The role of EphA3 in MM has not been previously investigated.

In this study, we first showed that EphA3 is highly overexpressed in MMECs. The over expression was detected with different approaches at mRNA and protein levels. Immunohistochemistry reaction in BM biopsies from MM patients allowed to clearly identify the EphA3 in ECs and in hematopoietic cells. Interestingly, we detected a lower EphA3 expression in ECs from MGUS but not in normal ECs. As MMECs were analyzed ˜30 days after harvesting from the BM, we suggest, tentatively, that changes in EphA3 gene and protein are stably acquired by these cells, with transition from MGUS (avascular phase) to MM (vascular phase) [35]. Perhaps genomic or epigenetic modifications could act on these genes/proteins. Therefore, epigenetic regulation, such as changes in gene methylation, may play a dominant role in upregulating the expression of EphA3 in neoplastic cells. This may be the result of already abnormal cell biology, also supported by the fact that EphA3 expression is not regulated by methylation of the promoter in normal tissue [18–29]. However, epigenetic characterization of MM, such as gene methylation and acetylation, has been slightly performed [32].

Interestingly, we observed a trend of increased EphA3 expression passing from untreated MM to refractory MM and to MM relapsed following anti angiogenic drugs such as lenalidomide and bortezomib. Moreover, it was published that EphA3 expression increased after s-thalidomide treatment in a MM cell line [33]. Taken together our and Liu data could help to give rationale for a combination therapy with IMiDs plus EphA3 inhibitor.

Angiogenesis is characterized by different steps such as cell proliferation, adhesion, migration and tube formation. The damage at any step of these processes will compromise the new vessel formation. We have demonstrated antiangiogenic effects of EphA3 knockdown (KD) *in vitro* at three levels: inhibition of adhesion, migration and tubular structure formation. This is in agreement with previously data that showed EphA3 silencing prevents spreading of LK63 cells on fibronectin surface [34].

Gene expression profiling and real time PCR showed that EphA3 KD inhibited some adhesion and pro angiogenic factor genes such as RYK, VEGFA, FLNA, CD248 [35–39]. We not observed change of the other Eph/Efn expression. The capacity of EphA3 to regulate these molecules in MMECs supports EphA3 as a regulator of angiogenesis.

We observed an interesting behavior of MMECs when analyzing quantitative expression of EphA3 in comparison to vessel formation capability. A Pearson correlation analysis indicated a significant and good correlation between EphA3 and length of arms in Matrigel assay. In conclusion, high-EphA3-expressing ECs (e.g. 9500 copies) showed higher propensity to form vessels *in vitro* as compared to low-EphA3-expressing ECs (e.g. 850 copies).

Interestingly, we obtained the same effects, a decreased migration and impaired capacity to form vessels *in vitro* also by treating MMECs with an antibody targeting EphA3 (chIIIA4 mAb). chIIIA4 mAb targets a site closely adjacent to the heterotetramerization site on the N-terminal of EphA3's extracellular domain adjacent to the ligand-binding site and has a high affinity for EphA3 [22, 29, 40]. The mechanism through which EphA3-specific antibody blocks MMECs adhesion and motility has not been investigated. However, we speculate that the anti-EphA3 antibody themselves may modulate EphA3 signaling by forcing it into a conformation that interferes with the signal transduction process; indeed, the antibody binding could modulate oligomerization and clustering of ligand or affect reverse signaling [41]. Moreover, we observed that EFNA5, a preferential ligand of EphA3, was expressed in a MM cell line (data not shown). MM cells and endothelium could interact via EphA3-EFNA5 binding. We speculate that anti EphA3 antibody could interfere in this binding by compromising endothelium-MM plasma cell communication. What happens as result of this binding interruption will object of next research. Further studies are needed to conclusively investigate the correct mechanisms of this function-modulating antibody.

The humaneered IIIA4 (KB004), is employed in a multi-center Phase 1/2 trial, (http://clinicaltrials.gov/ct2/show/NCT01211691) in patients with EphA3-positive hematologic neoplasms including Chronic Myelogenous Leukemia, Acute Myeloid Leukemia, Acute lymphoblastic leukemia, Myelodysplastic Syndrome who are refractory to, have failed, or have not received standard-of-care treatment (www.kalobios.com). Preliminary data show that, in one of leukemia patients, KB004 induces a response also targeting of stromal/fibrotic tumor microenvironment [21].

In summary, we have defined the biological role of EphA3 in MM angiogenesis. In addition, we have preliminary, demonstrated that EphA3 targeting by a specific antibody reduces the MM angiogenesis. Further studies are needed to evaluate whether EphA3 could represent a therapeutic target in patients affected by MM.

## MATERIALS AND METHODS

### Patients, endothelial cells (ECs)

Patients fulfilling the International Myeloma Working Group diagnostic criteria (International Myeloma Working Group, 2003) for active MM [*n* = 35, at first diagnosis (*n* = 23), in refractory phase to bortezomib or lenalidomide based chemotherapies (*n* = 3), in relapse after these therapies (*n* = 9)] and MGUS (*n* = 8) were studied. The MM patients (25 M and 10 F), aged 33–79 (median 66,2) years. The M component was IgG (*n* = 20), IgA (*n* = 15), and k or λ (*n* = 12). The MGUS patients (5 M and 3 F), aged 42–85 (median 60,8) years, were IgG (*n* = 6), IgA (*n* = 2), and k or λ (*n* = 5). Normal endothelial cells (ECs) were derived from 6 subjects with anemia due to iron or vitamin B_12_ deficiency (control subjects) [35]. The study was approved by the Ethics Committee of IRCCS-CROB (Prot 3 725; 7-2-2008) and all patients provided their informed consent in accordance with the Declaration of Helsinki. BM primary ECs from control subjects, from MM (MMECs) and from MGUS (MGECs) patients were obtained and cultured as described [42]. Briefly, separated mononuclear cells from BM aspirates were left to adhere to flask in complete medium [RPMI-1640 medium (Gibco, LifeTechnologies, USA) supplemented with 10% fetal calf serum (FCS, Gibco) and 1% glutamine (Gibco)] in culture conditions. Adherent cells were stromal cells. To isolate ECs, stromal cells were harvested and immunodepleted of macrophages and possible residual plasma cells by an incubation in CD14 plus CD38 monoclonal antibody (MoAb)–coated flasks (Immunotech, Coulter, Marseilles, France). Residual cells were incubated with magnetic microbeads (Dynal, Oslo, Norway) coated with Ulex europaeus agglutinin-1 (UEA-1; Sigma Chemical, St Louis, MO). Cells were recovered using a side-pool magnetic separation unit, transferred to 12-well plates in complete medium/well, and left to migrate to the plate surface and grow at 37°C under 5%CO_2_/95% humidified air. The MMEC antigene phenotype is described in Supplemental Experimental Procedures and data are reported in Table 2.

**Table 2:** Phenotypic characterization of MMECs

Antigens	% of positive cells
VEGFR2	95 ± 10
Tie2	84 ± 13
CD61	60 ± 20
CD144	82 ± 17
CD34	85 ± 19
FGFR2	41 ± 6
CD105	92 ± 15
CD38	15 ± 3
CD31	95 ± 18
CD62E	45 ± 7
CD138	-
CD14	-

### Absolute real-time PCR (RT-PCR)

RNA from MMECs, MGECs, normal ECs was extracted using Rneasy mini kit (Qiagen, Hilden, Germany). First strand cDNA was synthesized using random hexamers and transcriptor first strand cDNA kit (Roche, Molecular Biochemicals, Mannheim, Germany). Absolute RT-PCR was carried out using Taqman assay (Perkin-Elmer–Applied Biosystems, Massachusetts USA) in the Lightcycler 480 II (Roche). For the analysis of RYK, VEGF and FLNA mRNAs, SYBR Green I Master (Roche Applied Science) were used respectively, following the manufacturer's instructions. Cycling conditions and primers are listed in Supplemental Experimental Methods.

### Western blot (WB) and immunofluorescence-confocal laser scanning microscopy (IF)

Total protein lysates (80 μg) from MMECs, MGECs and normal ECs were blotted using 8% to 12% acrylamide gels, transferred to a polyvinylidene difluoride (PVDF) membrane; membranes were blocked with 5% non-fat dry milk in phosphate buffer saline (PBS) and incubated with primary antibodies for EphA3 (Abcam, Cambridge, UK) or for FLNA (Chemi-Con Corp., Nuremberg, Germany) and anti-β-actin (Sigma-Aldrich, St. Louis, MO, USA) overnight at 4°C. The membranes were then washed, incubated with appropriate horseradish peroxidase-conjugated secondary antibody (Cell Signaling Technology, Inc. Danvers, MA, USA). Immunoreactive bands were detected with enhanced chemiluminescence (Immun-Star HPR luminol, BioRad laboratories, USA) and ChemiDOC XRS system (BioRad, Segrate (MI), Italy), and quantified as optical density (OD) units by the Image Lab software (BioRad)

For confocal microscopy, 5×10^3^ MMECs, MGECs and normal ECs were cultured on fibronectin-coated chamber slides (LabTek, Nalge Nunc International, Naperville, IL, USA), fixed with paraformaldehyde, (Sigma Chemical Co., St. Louis, MO, USA), incubated with the anti-EphA3 antibody (AbCam), then goat anti-mouse Alexa Fluor 488 (Invitrogen, Carlsbad, CA, USA) was added and following washing they were incubated with TO-PRO-3 (Invitrogen). The cells were examined under a Leica TCS SP2 (Leica, Wetzlar, Germany) confocal laser scanning microscope using x63 objective lenses with either 1x or 2x zoom factors. A sequential scan procedure was applied during image acquisition of fluorophore. Confocal images were taken at 100-nm intervals through the z axis of the section. Images from individual optical planes and multiple serial optical sections were analyzed, digitally recorded, and stored as TIFF files using Adobe Photoshop software (Adobe Systems Inc. San Jose, CA, USA). The expression levels were showed as mean of Corrected Total Cell Fluorescence (CTCF) as Integrated Density – (Area of selected cell × Mean fluorescence of background readings).

### Fluorescence-activated cell sorting (FACS) analysis and immunohistochemistry

Three × 10^5^ cells/tube were incubated with a phycoerythrin (PE)-labelled anti-CD105 antibody (Beckman Coulter, Brea, CA, USA) in PBS. Cells washed and incubated with anti-EphA3 (AbCam) and isotype matched control antibodies. Fluorescein isothiocyanate (*FITC*)-anti-mouse IgG was added. At least 50 000 CD105 positive events per sample were analyzed using FACScantoII (Becton Dickinson, San Jose, CA, USA). The expression levels were showed as mean fluorescence intensity (MFI) of the antibody.

Formalin-fixed, 4 μm-thick BM sections of MM patients were stained with anti EphA3-specific monoclonal antibody SL2 (kindly provided by KaloBios Pharmaceuticals, San Francisco, CA, USA) and incubated with HRP conjugated anti-mouse secondary antibody (Dako Envision Plus). Staining was visualized using the liquid 3,3′-diaminobenzidine substrate-chromogen system (DakoCytomation, Carpinteria, CA, USA). Tissue sections were counterstained with Mayer's hematoxylin solution.

### Small interfering RNA (siRNA)

MMECs or MGECs or ECs (4 × 10^5^) were transiently transfected with 5 nM of a pool of EphA3–siRNA or control siRNA (Silencer Selecter siRNA Ambion, Lifetechnologies) or with the transfection reagent alone (Lipofectamine, RNAiMAX siRNA transfection reagent, Lifetechnologies) for 5 days and submitted to the followed functional studies.

### Treatment of MMECs with antibodies

MMECs were treated with anti EphA3 monoclonal antibody (chIIIA4 mAb) or an irrelevant Ab at 7.5 μg/ml or with Dulbecco's Modified Eagle Medium (DMEM; Gibco, Milan, Italy) alone and submitted to the followed functional studies.

### Functional studies

#### Viability and apoptosis

Viability was assessed by (3-(4,5-dimethylthiazol -2-yl)-5-(3-carboxymethoxyphenyl)-2-(4-sulfophenyl)-2H- tetrazolium) MTS assay, while the apoptotic cell rate by incubating treated cells with FITC–annexin V and Propidium iodide (Apoptosis detection kit, Becton Dickinson) followed by analysis on FACScantoII (Becton Dickinson).

#### Adhesion

One × 10^4^ siRNA MMECs were plated in DMEM on fibronectin-coated 96-well plates in triplicate for 30 min, fixed with 4% paraformaldehyde and quantified by the crystal violet assay at 595 nm in a Microplate Reader (Molecular Devices Corp., Sunnyvale, CA, USA).

#### Chemotaxis

Five × 10^4^ siRNA MMECs as above were tested in Boyden microchamber assay towards 1.5% serum medium with Vascular endothelial growth factor (VEGF 10 ng/ml, Sigma Chemical Co.) and fibroblast growth factor 2 (FGF-2; 10 ng/ml, Peprotech Inc., Rocky Hill, NJ, USA) as chemoattractants. After 8 h at 37°C, the migrated cells were fixed, stained and counted by the EVOS inverted microscope (Euroclone) at ×400.

#### Scratch wound healing assay

MMECs (2 × 10^4^ cells/well) were seeded in 96-well plates in complete DMEM and cultured to confluence. Confluent cell monolayer was then scraped with a yellow pipette tip to generate scratch wounds and washed twice with media to remove cell debris. Cells were incubated at 37°C for 24 h with the medium alone or containing anti EphA3 or isotype Ab control. Images were captured using a Nikon Eclipse TE2000-5 microscope. Four selected field of images were captured in each sample, and the wound areas were estimated by Nikon NIS-Elements computer software.

#### Angiogenesis on matrigel

MMECs treated as above were plated on Matrigel reduced growth factor (Becton Dickinson Biosciences, Bedford, MA) coated 48-well plates in serum-free medium (SFM) or in presence of anti EphA3 or isotype Ab after 18h, the skeletonization of the mesh was followed by measurement of mesh areas and vessel length in three randomly-chosen fields with the EVOS microscope at ×200.

### Gene expression profiling and microarray analysis

Total RNA from both EphA3 siRNA and Control siRNA MMECs was extracted using Rneasy mini kit (Qiagen). RNA quality was examined using the Agilent 2 100 bioanalyzer (Agilent Technologies UK Ltd., Cheshire, UK). For mRNA expression profiling, 300 ng total RNA were reverse transcribed and used for synthesis of cDNA and biotinylated cRNA according to the Illumina TotalPrep RNA Amplification Kit (Ambion, Cat. n. AMIL1791) protocol. For each sample, 750 ng of cRNA were hybridized for 17 hrs at 48°C on Illumina HumanHT-12 v4.0 BeadChips, containing 47,231probes (Illumina Inc.), according to the manufacturer's protocol and subsequently scanned with the Illumina HiScan. Data analyses were performed with GenomeStudio software (Illumina Inc.), by comparing all values obtained from siEphA3 vs Control siRNA MMECs values. Data was normalized with the quantile normalization algorithm, and genes were considered as detected if the detection *p-value* was lower than 0.05. Statistical significance was calculated with the Illumina DiffScore, a proprietary algorithm that uses the bead standard deviation to build an error model. Only genes with a DiffScore ≤–30 and ≥ 30, corresponding to a *p-value* of 0.001, were considered as statistical significant. Microarray data were submitted to Array Express under accession number E-MTAB-2519.

## SUPPLEMENTARY MATERIALS FIGURES AND TABLES




