# Supplementary material for: Epha3 acts as proangiogenic factor in multiple myeloma
Source: Oncotarget. 2017 Mar 10;8(21):34298–309. doi: 10.18632/oncotarget.16100 (PMC5470968; doi:10.18632/oncotarget.16100)
Supplement: Supplementary file 1 [file oncotarget-08-34298-s001.pdf]

Epha3 acts as proangiogenic factor in multiple myeloma

Supplementary Materials

Antigen phenotype

MMECs antigen phenotype was studied by fluorescence-activated cell sorting (FACS) analysis was performed with the fluorescein isothiocyanate (FITC)–conjugated UEA-1 lectin (Sigma) and FITC- or phycoerythrin (PE)–conjugated murine MoAbs to FGFR2, Tie2, CD61, CD144 (all from R&D system, Minneapolis, USA), CD62E, CD38 (all from BD Biosciences), CD105 (Beckman Coulter, Brea, CA, USA), CD14 antibodies. FITC or PE-conjugated IgG1 were used as isotype control antibodies. At least 10000 cells were read for each FACS labeling.

Absolute real-time PCR (RT-PCR)

Pre-defined assay reagents specific for EphA3 and ABL are reagents containing primers and probes (sequence unavailable from Applied Biosystems) optimised for use with the Lightcycler 480 II (Roche Molecular Biochemicals, Mannheim, Germany) and usually designed to span intron–exon boundaries.

A standard curve for both EphA3 and ABL was derived from the serial dilutions by a customary way (Real time Alert Nanogen Advanced Diagnostic Srl Turin, Italy). Knowing the copy number and concentration of plasmid DNA, the precise number of molecules added to subsequent real-time PCR runs can be calculated, thus providing a standard for specific cDNA quantification.

Real-time PCR runs were performed in 96-well optical plates in triplicate (each containing 1 × PCR master mix (Applied Biosystems), 0.2 pmol/μl of forward primer, reverse primer and labeled probe and 5 μl template DNA (either 100ng cDNA or plasmid DNA dilutions ranging from 1×10 to 1×10^5 in a final volume of 20 μl) for 40 cycles. Default cycle conditions were as follows; 10 minutes at 95ºC and 40 cycles of 30 s at 95ºC, 15 s at 60ºC, and 30 s at 72ºC. Expression represented as EphA3 copy number per 1×10^4 Abelson (ABL).

Sequences of sense and antisense primers used to amplify RYK, VEGF and FLNA cDNA were as follows: RYK forward 5′-AGGTGACAATGATGCTCACTGAA-3′, RYK reverse 5′ TGTGATGAAGACCTCGCAGCT-3′, VEGF forward 5′-AAGGAGGAGGGCAGAATCAT-3′, VEGF reverse 5′-CCAGGCCCTCGTCATTG-3′, FLNA forward 5′-CTGCATTTGGCGGAAAGTG-3′, FLNA reverse 5′-CTGCATTTGGCGGAAAGTG-3′. Cycling conditions of relative RT-PCR consisted of 10 minutes at 95°C, followed by 45 cycles of 15s at 95°C and 60 s at 60°C. Relative values of transcripts were calculated using the $2^{-\Delta\Delta Ct}$ method.

Supplementary Table 1: Gene analysis. See Supplementary_Table_1

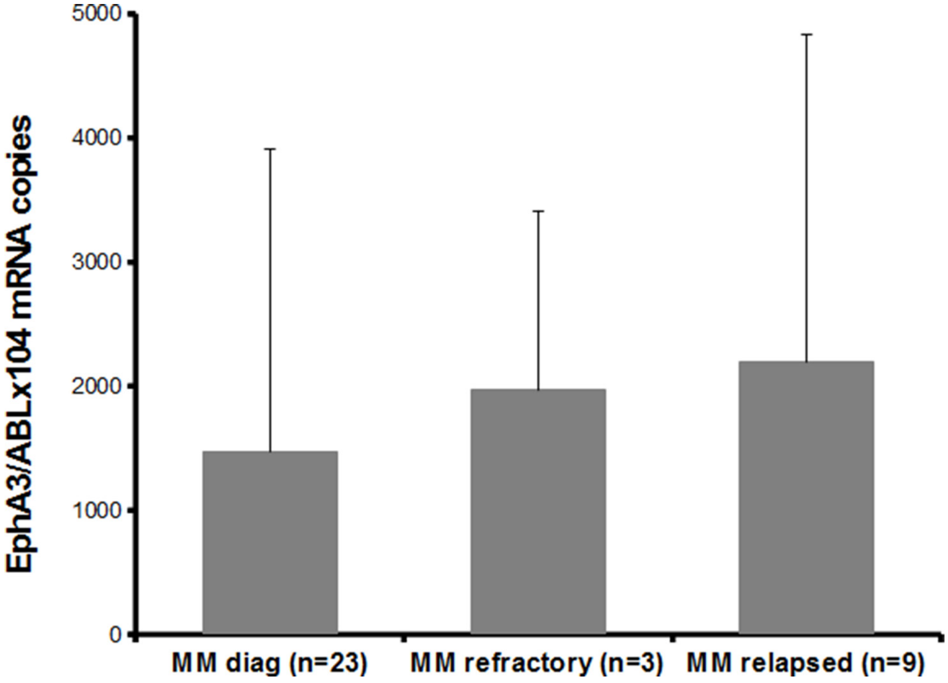

Supplementary Figure 1: EphA3 mRNA expression in ECs on the basis of MM stage. Absolute Real Time -PCR of EphA3 mRNA was performed. Data were reported as mean of EphA3 mRNA copies /10^4 *ABL* copies + SD of 23 at diagnosis and 3 refractory and 9 relapsed MM subjects, respectively.

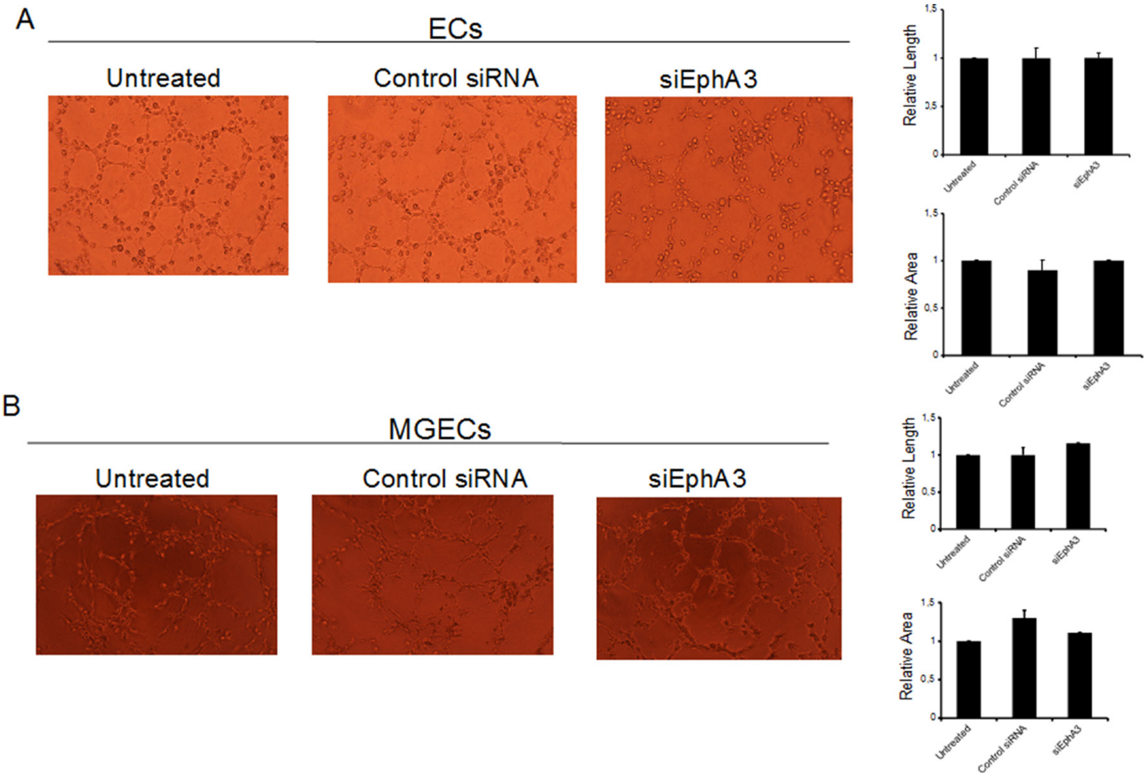

Supplementary Figure 2: Effects of siEphA3 in ECs and MGECs on *in vitro* tube formation. ECs (**A**) and MGECs (**B**) silenced for EphA3 or control siRNA or untreated were tested for angiogenesis on Matrigel (quantification of vessel length and areas in the right panels). Images were analised by the EVOS image software. Matrigel original magnification X10 for all panels. Data are means + SD of 3 MGEC patients and 3 ECs.

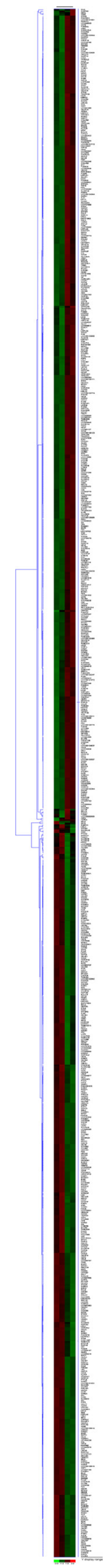

Supplementary Figure 3: Heat map of modulated genes across siEphA3 MMECs and Control siRNA-MMECs. The heatmap of all the differentially expressed genes was generated using the dChip software.
